# Race, gender, class, and sexual orientation: intersecting axes of inequality and self-rated health in Canada

**DOI:** 10.1186/1475-9276-10-3

**Published:** 2011-01-17

**Authors:** Gerry Veenstra

**Affiliations:** 1 Department of Sociology, University of British Columbia, Vancouver, British Columbia, Canada

## Abstract

**Background:**

Intersectionality theory, a way of understanding social inequalities by race, gender, class, and sexuality that emphasizes their mutually constitutive natures, possesses potential to uncover and explicate previously unknown health inequalities. In this paper, the intersectionality principles of "directionality," "simultaneity," "multiplicativity," and "multiple jeopardy" are applied to inequalities in self-rated health by race, gender, class, and sexual orientation in a Canadian sample.

**Methods:**

The Canadian Community Health Survey 2.1 (N = 90,310) provided nationally representative data that enabled binary logistic regression modeling on fair/poor self-rated health in two analytical stages. The additive stage involved regressing self-rated health on race, gender, class, and sexual orientation singly and then as a set. The intersectional stage involved consideration of two-way and three-way interaction terms between the inequality variables added to the full additive model created in the previous stage.

**Results:**

From an additive perspective, poor self-rated health outcomes were reported by respondents claiming Aboriginal, Asian, or South Asian affiliations, lower class respondents, and bisexual respondents. However, each axis of inequality interacted significantly with at least one other: multiple jeopardy pertained to poor homosexuals and to South Asian women who were at unexpectedly high risks of fair/poor self-rated health and mitigating effects were experienced by poor women and by poor Asian Canadians who were less likely than expected to report fair/poor health.

**Conclusions:**

Although a variety of intersections between race, gender, class, and sexual orientation were associated with especially high risks of fair/poor self-rated health, they were not all consistent with the predictions of intersectionality theory. I conclude that an intersectionality theory well suited for explicating health inequalities in Canada should be capable of accommodating axis intersections of multiple kinds and qualities.

## Background

Sizeable health inequalities by race [1,2], gender [3,4] and class [5] have been recorded in Canada. Consistent with traditional sociological understandings of social inequality, these axes of inequality have for the most part been considered individually, with researchers only considering potential interconnectedness when investigating whether class mediates associations between race and health or gender and health. Whether class influences health differently for visible minority Canadians and White Canadians or race influences health differently for men and women, for example, has not yet been investigated. When statistical interactions such as these *have *received analytical attention - for example, whether class influences health differently for Canadian men and women [3] - they have not been adequately theorized. Intersectionality theory, an influential theoretical tradition inspired by the feminist and antiracist traditions, demands that inequalities by race, gender, and class (and sexuality as well) be considered in tandem rather than distinctly. This is because these fundamental axes of inequality in contemporary societies are considered to be intrinsically entwined; they mutually constitute and reinforce one another and as such cannot be disentangled from one another. Intersectionality theory presents a new way of understanding social inequalities that possesses potential to uncover and explicate previously unknown health inequalities. This paper describes the results of an original empirical investigation of the degree to which the self-rated health of Canadians varies by race, gender, class, and/or sexual orientation in ways that are consistent with predictions of intersectionality theory. The remainder of this background section describes some of the central principles of this theoretical tradition followed by a description of the analytical strategy used to apply these principles in an empirical investigation of inequalities in self-rated health in Canada.

### Intersectionality theory

In the forward to a recent book on new theories and methods for studying race, class, and gender, Lynn Weber [6] describes how American women of color in the 1970s and early 1980s, many from working class backgrounds, came to critique the patriarchy tradition within gender studies for privileging gender over race and class (and subsequently critiqued the stratification tradition for privileging class over gender and race, etc.). They argued that these axes of inequality are in fact analytically inseparable, and that "the multidimensionality and interconnected nature of race, class, and gender hierarchies were especially visible to those who faced oppression along more than one dimension of inequality" [6:xii]. These scholars envisioned axes of inequality pertaining to gender, race, and class that *intersect *with one another, i.e., that are interlocked, dependent upon one another, and mutually constituted [7]. Power relationships along the lines of gender, race, and class were thought to be mutually defining and mutually reinforcing rather than analytically distinct systems of oppression, together forming a "matrix of domination" [8]. By the mid-1980s, lesbians of color had bridged the gap between gay and lesbian studies and the growing body of race, gender, and class research that had to that point ignored heterosexism [6], and axes of inequality pertaining to national origin, citizenship status, religion, disability, and age also received some attention. The contributions of these various scholars gave rise to what is now known as "intersectionality theory." Landry [9] notes, however, that intersectionality theory does not provide a set of propositions that together form an explanation; rather, intersectionality theory currently consists of a loose set of principles or assumptions that are being applied and tested by many researchers in a variety of contexts.

Founded upon analyses of relations of power in general and inspired by theories of racism, patriarchy, classism, and heterosexism in particular, in American intersectionality discourse the disadvantaged groups along the inequality axes of race, gender, class, and sexual orientation are assumed to be visible minorities from various backgrounds (especially African Americans), women, members of the lower and working classes, and gays, lesbians, and bisexuals. These comprise implicit intersectionality assumptions of "directionality."

Intersectionality theorists argue that our identities based on race, gender, class, and sexuality accompany us in every social interaction [7]. The principle of "simultaneity" maintains that all of the axes and their corresponding identities should be incorporated into social analyses.

"Race, class and gender may all structure a situation but may not be equally visible and/or important in people's self-definitions... This recognition that one category may have salience over another for a given time and place does not minimize the theoretical importance of assuming that race, class and gender as categories of analysis structure all relationships" [7:560-1].

That is, while some axes and identities may be more pertinent to a specific social context or outcome than are others, simultaneity implies that a social researcher should never discard an axis of inequality before investigating its potential relevance for the problem at hand.

Intersections between axes are thought to create complex social locations that are more central to the nature of social experiences than are any of the axes of inequality considered singly.

"People experience race, class, gender and sexuality differently depending upon their social location in the structures of race, class, gender and sexuality. For example, people of the same race will experience race differently depending upon their location in the class structure as working class, professional managerial class or unemployed; in the gender structure as female or male; and in structures of sexuality as heterosexual, homosexual or bisexual" [10:326-7].

Thus "multiplicativity" should supplant additivity [10]. Racism x sexism x classism x sexism should replace racism + sexism + classism + sexism [11,12]. A lower-class Black lesbian is necessarily all of these things, and their mutual manifestation represents a unique state of being and a unique set of social experiences and structural constraints.

"Race, class, gender and sexuality are not reducible to individual attributes to be measured and assessed for their separate contribution in explaining social outcomes, an approach that Elizabeth Spelman calls "pop-bead metaphysics," where a woman's identity consists of the sum of parts neatly divisible from one another. The matrix of domination seeks to account for the multiple ways that women experience themselves as gendered, raced, classed and sexualized" [10:327].

Experiences of gender are racialized, sexualized, and classed; experiences of class are gendered, racialized, and sexualized, etc.

From the abovementioned principles of directionality, simultaneity, and multiplicativity arise new versions of double jeopardy and triple jeopardy, renamed "multiple jeopardy" by Deborah King [11], wherein disadvantaged identities experienced in tandem are seen to result in inordinate, i.e., even more than additive, amounts of disadvantage. Thus complex social locations comprised of disadvantaged identities held in tandem are thought to lead to multiplicative disadvantage; that is, combinations of these identities are thought to have an aggravating rather than a simply cumulative or mitigating effect. In addition, because of the relational nature of intersectional theories, some complex locations, such as the one inhabited by wealthy heterosexual White men, in turn experience multiplicative advantage.

Despite the immense popularity of intersectionality theory in humanities and social sciences circles and the large and growing body of intersectionality research that includes applications of both qualitative and quantitative methodologies, very little quantitative research has explicitly applied intersectionality theory to health outcomes. However, many health determinants researchers have unintentionally addressed simultaneity and multiplicativity by identifying two-way statistical interactions between axes of inequality in regression modeling. In Canada, Zheng Wu and colleagues [2] identified interactions between race and socioeconomic status for depression. In the United States, Ostrove and colleagues [13] identified interactions between socioeconomic status and race as predictors of self-rated health and depression, Nomagushi [14] found interactions between race and gender on the effect of marital dissolution on depression, and Read and Gorman [15] determined that the gender gap in health differs widely by racial/ethnic group. But only a few quantitative studies have explicitly studied illness states associated with complex social positions arising from intersections between three axes of inequality [16-19], none of them Canadian, and no studies have studied intersections between all four of the primary axes of inequality of intersectionality theory. Given the seeming complicity of all of race [2,20-23], gender [3,4,24], class [5,25-29], and sexual orientation [30-33] in North American health inequalities, this lack of attention to health inequalities that accrue to multiple combinations of inequality identities represents an important gap in the health determinants literature.

### Analytical strategy

Modeling the main effects of inequality identities (additivity) and then statistical interactions between them (multiplicativity) in multivariate regression models on health can establish whether two-way or three-way statistical interactions (intersections) between axes of inequality contribute to explaining variability in health above and beyond the additive approach to health inequalities that currently dominates health determinants research. This paper uses a two-stage analytical strategy, the first additive and the second multiplicative, applied to a large representative survey dataset from Canada in order to investigate health outcomes associated with intersections between race, gender, class, and sexual orientation.

First, the strength and direction of the main effects in additive regression models such as Race + Gender + Class + Sexual Orientation = Health addresses the principles of simultaneity and directionality. Simultaneity suggests that all four identities will make significant contributions to these models before and/or after controlling for one another while directionality implies that non-Whites, women, lower-class people, and non-heterosexuals will manifest the poorer health outcomes.

Second, simultaneity and multiplicativity imply that the inequality identities should interact meaningfully with one another as predictors of health, that is, statistical interactions between the inequality variables of race, gender, class, and sexual orientation should manifest significant effects above and beyond their main effects in the abovementioned additive models. The existence of interactions speaks to multiplicativity. The qualities of the interactions themselves speak to multiple jeopardy and directionality. At least three multiplicative scenarios are possible for a given statistical interaction: 1. two or more axes of inequality manifest directions of some kind or other in additive models and then display an aggravating effect in the interaction between them, 2. two or more axes manifest given directions in additive models and then display a mitigating effect in their interaction, and 3. an interaction manifests itself between two or more axes but not all of the axes display independent effects in additive models. Aggravating effects support the assumption of multiple jeopardy and reinforce the directionality identified in the additive models whereas non-aggravating effects run contrary to the assumption of multiple jeopardy and complicate directionality. Finally, contributions to predicted variability in the models address multiplicativity by providing an indication of the "value added" of the statistical interactions; comparisons of R^2 ^values between regression models with and without the cross-product terms can be used to assess the magnitude of their contributions to explaining variability in health above and beyond the contributions of the main effects.

## Methods

### Survey sample

The *Canadian Community Health Survey 2.1 *dataset was collected by Statistics Canada in 2003. The target population for this cross-sectional survey was all persons 12 years of age and older residing in Canada, excluding individuals living on Indian Reserves and on Crown Lands, institutional residents, fulltime members of the Canadian Armed Forces, and residents of some remote regions. Sampling considered province or territory and health region of residence and applied three sampling frames (a multistage stratified cluster design in an area frame, a list frame of telephone numbers, and a random digit dialing frame) to select the sample of households. One person was chosen randomly from each household to complete the survey. A total of 134,072 usable responses were obtained, representing a national response rate of 80.7%. Final person estimation weights were provided by Statistics Canada.

This investigation focuses on survey respondents who were aged 25 and older at the time of the survey. Table 1 describes socio-demographic characteristics of this sample of 109,967 respondents. The logistic regression models were applied to the 90,310 respondents with valid information for the age, race, gender, education, household income, sexual orientation, and self-rated health variables. Household income (N = 15,481) and sexual orientation (N = 7,676) were the main contributors to the loss of cases from listwise deletion. In comparison with the working sample, the sample of missing cases was older, poorer, and less educated on average and contained proportionately more widows, non-Whites, and adult immigrants to Canada.

**Table 1:** Characteristics of the sample (weighted data)

**Variable**	**Categories**	**Distribution**
Gender	male	53,578 (48.7%)
	female	56,389 (51.3)
Marital status	married	68,255 (62.2%)
	living common-law	10,356 (9.4)
	widowed	6,916 (6.3)
	separated	3,048 (2.8)
	divorced	6,049 (5.5)
	single (never married)	15,135 (13.8)
Age	aged 25 - 34	21,639 (19.7%)
	aged 35 - 44	27,611 (25.1)
	aged 45 -54	23,839 (21.7)
	aged 55 - 64	17,155 (15.6)
	aged 65 and older	19,732 (17.9)
Sexual orientation	heterosexual	100,803 (98.5%)
	homosexual	945 (0.9)
	bisexual	543 (0.5)
Educational attainment	less than secondary	21,582 (20.1%)
	secondary graduate	26,463 (24.7)
	community college; technical school; some university (no degree)	36,496 (34.0)
	bachelor's degree	15,466 (14.4)
	post-bachelor degree	7,237 (6.7)
Household income	< $10,000	2,291 (2.4%)
	$10,000 - 19,999	8,130 (8.6)
	$20,000 - 29,999	9,664 (8.8)
	$30,000 - 39,999	10,409 (11.0)
	$40,000 - 49,999	9,862 (10.4)
	$50,000 - 59,999	9,708 (10.3)
	$60,000 - 79,999	16,108 (17.0)
	$80,000 or more	28,313 (30.0)
Race	White	90,864 (85.5%)
	Chinese	3,676 (3.5)
	South Asian (e.g., East Indian, Pakistani, Sri Lankan)	2,758 (2.6)
	Black	1,617 (1.5)
	Aboriginal (North American Indian, Métis and Inuit)	1,028 (1.0)
	Filipino	998 (0.9)
	Latin American	848 (0.8)
	Southeast Asian (e.g., Cambodian, Indonesian, Laotian, Vietnamese)	594 (0.6)
	Arab	523 (0.5)
	West Asian (e.g., Afghan, Iranian)	311 (0.3)
	Korean	284 (0.3)
	Japanese	204 (0.2)
	other	1,509 (1.4)
	multiple origins	1,108 (1.0)
Immigrant status	immigrated to Canada as adult (aged 18 and older)	18,260 (17.2%)
	immigrated to Canada as child (under 18)	6,204 (5.8)
	born in Canada	81,834 (77.0)
Self-rated health	poor	3,361 (3.1%)
	fair	10,865 (9.9)
	good	33,919 (30.9)
	very good	38,138 (34.7)
	excellent	23,600 (21.5)

### Survey measures

Survey respondents were asked the following question: "People living in Canada come from many different cultural and racial backgrounds. Are you: White? Chinese? South Asian (e.g., East Indian, Pakistani, Sri Lankan)? Black? Filipino? Latin American? Southeast Asian (e.g., Cambodian, Indonesian, Laotian, Vietnamese)? Arab? West Asian (e.g., Afghan, Iranian)? Japanese? Korean? Aboriginal (North American Indian, Métis or Inuit)? Other - specify." The interviewer was instructed to read all of the possible responses and record all of them that applied. Due to small sample sizes for some responses this variable was recoded as follows: Aboriginal, Asian (combining the Chinese, Korean and Japanese categories), Black, South Asian, and White, as well as a residual category created by combining the remaining categories, including the original "other" category, into a single un-interpretable category labeled "other."

Highest educational attainment and household income were used to assess class standing. Statistics Canada asked a series of survey questions pertaining to educational attainment to generate the education variable described in Table 1. To assess household income, respondents were asked: "What is your best estimate of the total income, before taxes and deductions, of all household members from all sources in the past 12 months?" Follow-up questions determined the range within which their household income fell for those respondents unable or unwilling to provide a precise dollar value.

Sexual orientation was assessed as follows: "Do you consider yourself to be: Heterosexual? (sexual relations with people of the opposite sex); Homosexual, that is lesbian or gay? (sexual relations with people your own sex); Bisexual? (sexual relations with people of both sexes)" Approximately 0.6% of women and 0.5% of men self-reported as bisexual and 0.7% of women and 1.2% of men self-reported as homosexual, values that are slightly lower than numbers reported by similar studies in the United States [32], Australia [34], and the Netherlands [35] where approximately 2-3% of the general population reported being homosexual or bisexual.

Global self-rated health, a variable known to encompass both physical and mental well-being and to reliably predict other, more objective, measures of health [36] as well as mortality [37], was assessed as follows: "I'll start with a few questions about your health in general. In general, would you say your health is: Excellent? Very good? Good? Fair? Poor?"

### Regression modeling

Self-rated health was dichotomized so that fair and poor responses were contrasted with good, very good, and excellent responses and binary logistic regression modeling was then used to predict fair/poor health. Each nominal independent variable in a regression model was treated as a set of dummy variables with one (missing) dummy variable serving as the reference. Because the N for a reference category should be large in order to provide a stable reference point, "White" was assigned the reference category for race and "heterosexual" was assigned the reference category for sexual orientation. In addition, "male" was assigned the reference category for gender and "postgraduate degree" was assigned the reference category for education. This strategy facilitated ready interpretation of how the other identities fare relative to what are generally considered the more privileged identities in Canadian society. Nagelkerke pseudo R^2^, a rough measure of the proportion of variability explained by a logistic regression model, was presented for each additive model.

Introducing cross-product terms to hierarchically well-ordered models is a common approach to investigating statistical interactions in the context of logistic regression [38]. Alpha was set at 0.05 with regards to the contributions of main effect terms in additive logistic regression models but at 0.10 for the interaction terms because of the lesser power of tests of significance for interactions in general (some of the variation in the dependent variable explained by the interaction may be already captured by the main effect test, measurement error in the individual factors becomes compounded in an interaction term, etc.).

The logistic regression models were implemented in SPSS 15.0. Because the sampling design for the CCHS 2.1 was complex, the 500 bootstrapping weights and BOOTVAR program created for the CCHS 2.1 by Statistics Canada were used to generate more reliable variance estimates and thus more reliable tests of significance and confidence intervals for individual variables within regression models. Due to the limitations of BOOTVAR, results from omnibus tests of significance for categorical variables and interaction terms comprised of sets of dummy variables and Model Chi-square tests of significance for logistic regression models in their entirety could not be generated.

## Results

### Additive models

Table 2 describes the key features of a set of additive binary logistic regression models on self-rated health. With regards to race, Table 2 indicates that Aboriginals, Asians, and South Asians were significantly more likely than Whites to report fair/poor self-rated health. The women of the sample were slightly more likely than the men to report fair or poor self-rated health, controlling for age, but upon additionally controlling for the other inequality variables gender was not significantly related to self-rated health. Educational attainment and household income were both significantly associated with self-rated health, in the expected directions, before and after controlling for the other variables. Finally, self-identified bisexual respondents were more likely than heterosexuals to report fair or poor self-rated health, holding age constant, although the association weakened to the point of non-significance after controlling for the other inequality variables. The decline in effect size for Aboriginal identity compared to White identity from Model I to Model V was mostly due to differences in education and income whereas the declines in effect sizes for female compared to male identity and bisexual orientation compared to heterosexual orientation were primarily due to differences in income (results not shown).

**Table 2:** Binary logistic regression models on fair/poor self-rated health

	**Model I**		**Model II**		**Model III**		**Model IV**		**Model V**	
	***OR***	***95% CI***	***OR***	***95% CI***	***OR***	***95% CI***	***OR***	***95% CI***	***OR***	***95% CI***
Race										
Aboriginal	2.562***	[2.048 .. 3.206]	----		----		----		1.707***	[1.364 .. 2.136]
Asian	1.426**	[1.131 .. 1.798]	----		----		----		1.392**	[1.093 .. 1.772]
Black	1.186	[0.803 .. 1.753]	----		----		----		1.008	[0.670 .. 1.517]
South Asian	1.313	[0.997 .. 1.729]	----		----		----		1.337*	[1.010 .. 1.771]
other	1.570***	[1.313 .. 1.871]	----		----		----		1.490***	[1.239 .. 1.792]
White	1.000		----		----		----		1.000	
Gender (female)	----		1.177***	[1.108 .. 1.251]	----		----		1.051	[0.986 .. 1.120]
Educational attainment										
less than secondary	----		----		2.931***	[2.312 .. 3.713]	----		3.018***	[2.386 .. 3.817]
secondary graduate	----		----		2.033***	[1.606 .. 2.576]	----		2.090***	[1.655 .. 2.640]
cc/ts/some university	----		----		1.810***	[1.430 .. 2.282]	----		1.872***	[1.485 .. 2.360]
bachelor degree	----		----		1.192	[0.923 .. 1.530]	----		1.187	[0.923 .. 1.525]
postgraduate degree	----		----		1.000		----		1.000	
Household income	----		----		0.458***	[0.430 .. 0.487]	----		0.473***	[0.444 .. 0.504]
Sexual orientation										
homosexual	----		----		----		1.172	[0.858 .. 1.601]	1.239	[0.903 .. 1.700]
bisexual	----		----		----		1.955**	[1.293 .. 2.954]	1.534	[0.997 .. 2.361]
heterosexual	----		----		----		1.000		1.000	
*Nagelkerke R^2^*	0.088		0.084		0.141		0.084		0.144	

Age controlled in all models; N = 90,310 for all models; *p < .05, **p < .01, ***p < .001 in two-tailed tests of significance.

Comparisons of odds ratios and Nagelkerke R^2 ^values indicate that education and income followed by race were the strongest predictors of self-rated health. Education and income were also implicated in some of the "hidden" explained variability in the regression models (results not shown). Regarding the overall contributions of the main effects to predicted variability in health, as a set the five inequality variables produced an increase in Nagelkerke R^2 ^of 0.061 over the regression model on self-rated health containing age alone.

In summary, the additive models of Table 2 described poorer health outcomes for bisexual respondents, non-White respondents, and respondents of lower class standing. The health effects of gender were minimal and the health scores of homosexuals did not differ significantly from those of heterosexuals. Class was the strongest distinct predictor of health of the four axes of inequality. With regards to the principle of simultaneity, these results suggest that sexual orientation, race, and class are especially relevant intersectionality axes of inequality in this national context, with directions that point to the negative health experiences of bisexuals, members of lower classes, and Canadians claiming Aboriginal, Asian, or South Asian identities in particular.

### Multiplicative models

**Figure 1:**
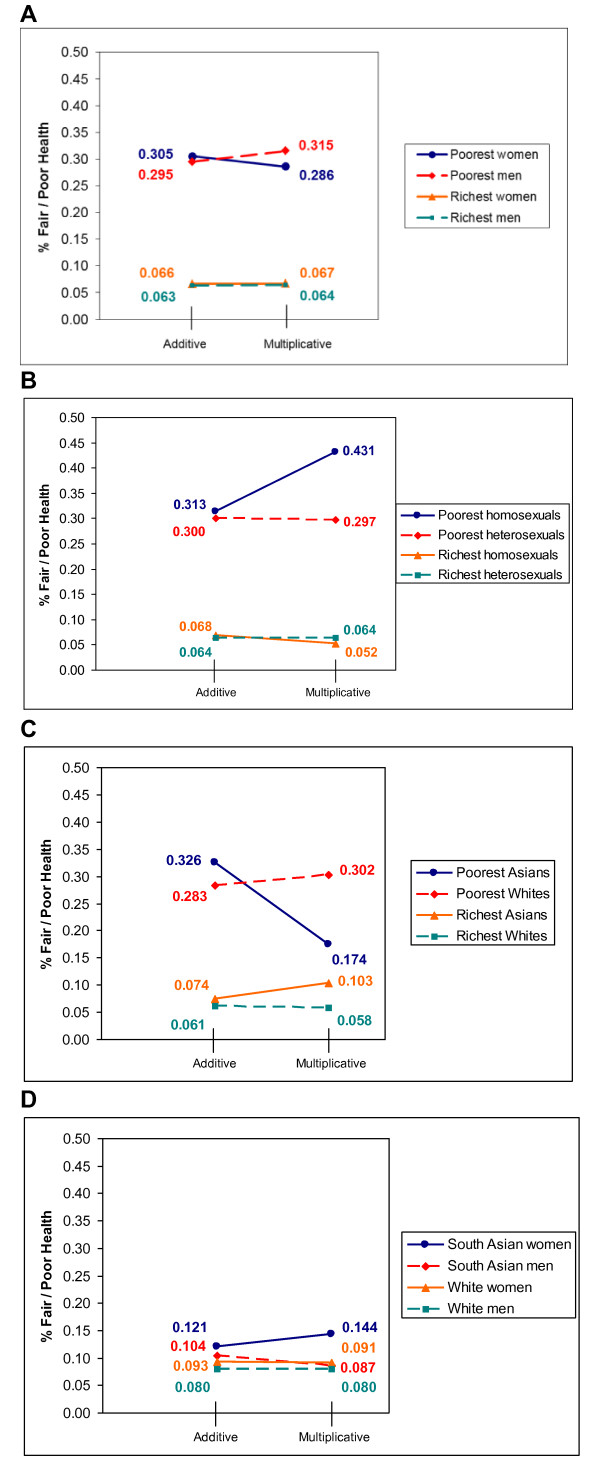
**Predicted Probabilities of Fair/Poor Self-rated Health**. A: Income by gender; B: Income by sexual orientation; C: Income by race; D: Race by gender.

Two-way and three-way interactions between the five inequality variables were individually added to the final additive model of Table 2. Interactions that included education and income, the two indicators of class, were not considered. Insufficiently large cell sizes precluded investigation of the two-way interaction between race and sexual orientation and the three-way cross-product terms that included sexual orientation and necessitated use of a dichotomized version of education (has a university degree or not) in the two-way and three-way interactions that included education and race. Table 3 contains odds ratios and p-values for the statistically significant interactions. Figure 1 depicts predicted probabilities for statistically significant interactions; the probabilities labeled "additive" were generated from additive models that did not contain any interaction terms and the probabilities labeled "multiplicative" were generated from models that additionally contained the interaction terms of interest. These visual depictions of predicted probabilities aid in determining whether aggravating effects (multiplicative advantage or disadvantage) or non-aggravating effects (such as mitigating effects) pertained to the multiplicative scenarios.

**Table 3:** Statistical interactions on self-rated health

**1. Income by gender**			
Female	OR income	=	0.502 (comparison with 0.439 produces p = .011)
Male	OR income	=	0.439
**2. Education by gender**			
Female	OR less than post-secondary	=	2.428 (comparison with 3.645 produces p = .090)
	OR secondary graduate	=	1.944 (comparison with 2.110 produces p > .100)
	OR cc/ts/some university	=	1.600 (comparison with 2.116 produces p > .100)
	OR bachelor degree	=	1.166 (comparison with 1.145 produces p > .100)
	OR postgraduate degree	=	1.000
Male	OR less than post-secondary	=	3.645
	OR secondary graduate	=	2.110
	OR cc/ts/some university	=	2.116
	OR bachelor degree	=	1.145
	OR postgraduate degree	=	1.000
**3. Education by sexual orientation**			p > .100 in all comparisons
**4. Income by sexual orientation**			
Homosexual	OR income	=	0.306 (comparison with 0.474 produces p = .050)
Bisexual	OR income	=	0.605 (comparison with 0.474 produces p > .100)
Heterosexual	OR income	=	0.474
**5. Education^1 ^by race**			p > .100 in all comparisons
**6. Income by race**			
Aboriginal	OR income	=	0.442 (comparison with 0.444 produces p > .100)
Asian	OR income	=	0.804 (comparison with 0.444 produces p < .001)
Black	OR income	=	0.731 (comparison with 0.444 produces p > .100)
South Asian	OR income	=	0.335 (comparison with 0.444 produces p > .100)
other	OR income	=	0.696 (comparison with 0.444 produces p = .004)
White	OR income	=	0.444
**7. Sexual orientation by gender**			p > .100 in all comparisons
**8. Race by gender**			
Female	OR Aboriginal	=	1.628 (comparison with 1.818 produces p > .100)
	OR Asian	=	1.597 (comparison with 1.185 produces p > .100)
	OR Black	=	1.038 (comparison with 0.972 produces p > .100)
	OR South Asian	=	1.808 (comparison with 1.031 produces p = .050)
	OR other	=	1.667 (comparison with 1.323 produces p > .100)
	OR White	=	1.000
Male	OR Aboriginal	=	1.818
	OR Asian	=	1.185
	OR Black	=	0.972
	OR South Asian	=	1.031
	OR other	=	1.323
	OR White	=	1.000
**9. Income by gender by race**			p > .100 in all comparisons
**10. Education^1 ^by gender by race**			p > .100 in all comparisons

1. Education in dichotomous form (has university degree)

Neither of the three-way interactions had a statistically significant effect on self-rated health. However, each of gender, race, and sexual orientation manifested significant two-way interactions with class and gender interacted significantly with race (Table 3). Consider first the interaction between gender and income. Table 3 indicates that income manifested a stronger association with self-rated health among men (OR = 0.439) than among women (OR = 0.502) and that the ratio of the two odds ratios differed significantly from 1 (p = .011). Figure 1A depicts additive predicted probabilities of 0.305 for the poorest women, 0.295 for the poorest men, 0.066 for the richest women and 0.063 for the richest men. These predicted probabilities reflect the weak gender effect and strong income effect evident in the final additive model of Table 2. The plot also contains predicted probabilities from a multiplicative model incorporating the interaction between gender and income. Here we see that the predicted probability of fair/poor health among the poorest women (0.286) was somewhat lower than we would expect from an additive perspective. The interaction between gender and income on self-rated health therefore represents a mitigating effect for lower-class women.

The marked change for the worse in risk of fair/poor health from the additive model to the multiplicative model for poor homosexuals depicted in Figure 1B is an aggravating effect in the form of multiplicative disadvantage experienced by poor homosexuals. The self-rated health of Asians was much less influenced by income than was the self-rated health of Whites; in particular, the risk of self-rated health depicted in Figure 1C was much lower than expected for the poorest Asians, a mitigating effect. Finally, South Asian women were more likely than White women to report fair/poor self-rated health while South Asian men were no more likely than White men to do so (Table 3). The increase in risk of fair/poor self-rated health among South Asian women from the additive model to the multiplicative model depicted in Figure 1D seemingly represents a case of multiplicative disadvantage experienced by South Asian women.

Adding all of the two-way cross-product terms to the final model of Table 2 produced an increase of 0.007 in the Nagelkerke R^2^. Two-way interactions between the four axes of inequality therefore contributed less than one percent predicted variability in self-rated health.

In summary, each of the four axes of inequality interacted significantly with at least one other, suggesting that all four axes belong to the pantheon of intersectionality axes of inequality that contribute to health inequalities in Canada. The only instances of multiplicative disadvantage pertained to poor homosexuals and to South Asian women who were at an especially high risk of fair/poor self-rated health. Mitigating effects pertained to lower class women and to poorer Asians who were less likely to report fair/poor health than expected. Lastly, the multiplicative models contributed relatively little to overall predicted variability in self-rated health over and above the contribution of the full additive model.

## Discussion

From the perspective of intersectionality theory, by focusing on a subset of the inequality identities or by treating multiple axes of inequality as distinct rather than intersected processes, a social researcher is in danger of misunderstanding the nature of social experiences and identities manifested in specific contexts and thus in danger of producing results and interpretations that are as misleading as they are incomplete. If this is true then much of the literature on health effects of inequalities pertaining to race, gender, class, and/or sexual orientation is incomplete, and some of it may even be misleading.

The Canadian Community Health Survey dataset is especially well suited to investigating the applicability of intersectionality theory to health disparities in Canada. It is the first and only Statistics Canada survey dataset to assess sexual orientation, distinguishing between bisexuals, homosexuals, and heterosexuals, and unlike most Canadian survey datasets it is large enough to produce a multi-category measure of race. The analysis described herein is therefore unique by virtue of its consideration of intersections between all four key inequality axes of intersectionality theory, its consideration of bisexual identities as well as homosexual and heterosexual identities, and its consideration of racialized identities such as Aboriginal, Asian, and South Asian as well as Black and White. In addition, the application of central principles of intersectionality theory to Canada, close neighbor to the United States, can contribute to future speculation about the portability of intersectionality assumptions across borders. Cross-contextual comparisons are essential in light of the fact that institutionalized race relations, gender relations, etc. are historically and contextually specific [39]. However, several important limitations of the study require acknowledgment. The validity of the sexual orientation survey question is of some concern. The small percentage of people who chose a non-heterosexual orientation in general suggests that many survey respondents may have been unwilling to reveal a historically stigmatized identity to interviewers. The especially small percentages of people reporting a non-heterosexual orientation in several of the non-White groups speaks to cultural differences in professing stigmatized non-heterosexual orientations, a knotty measurement problem for any study that seeks to investigate intersections between sexual orientation and race. Lastly, by virtue of excluding Indian Reserves from the sampling process the survey sample does not represent on-reserve Aboriginal people in Canada who are known to have even poorer health than off-reserve Aboriginal Canadians [40].

The intersectionality principle of simultaneity maintains that all four axes of inequality should be considered in an analysis while the principle of multiplicativity maintains that intersections between axes should overshadow or supplant the individual axes themselves in their effects. Although we carry our identities into every social situation, not all of them are necessarily salient in or relevant to a particular encounter [7]. Even so, race, gender, class, and sexual orientation all manifested independent relationships with health at the additive stage of my analysis and each of the four axes intersected meaningfully with at least one other axis, suggesting that all four of these intersectionality axes of inequality were operative for better or for worse in many of the social situations encountered by survey respondents in their everyday lives. In short, the principles of simultaneity and multiplicativity founded upon the inequality foursome of race, gender, class, and sexual orientation appear to be relevant for disparities in health in Canada.

The intersectionality assumption of multiple jeopardy maintains that meaningful intersections manifest multiplicative - inordinate amounts of - disadvantage or advantage. While two intersections were to indeed to the further detriment of certain complex social locations, i.e., of poor homosexuals and South Asian women, two demonstrated a mitigating quality for certain complex locations, i.e., for lower class women and poor Asian Canadians. Many other possible interactions were not large or statistically significant. It therefore appears that, with regards to self-rated health in Canada at least, multiple jeopardy can be more *or *less than (or most often simply equal to) cumulative double or triple jeopardy. This multiplicity of multiplicative possibilities demands a kind of conceptual fluidity that is not accommodated by the principle of multiple jeopardy as it is depicted it in the introduction to this paper.

Bart Landry [9] argues that while the notion of oppression is useful and undoubtedly reflects real experiences, for intersectionality theory to realize its full potential in social research it must accommodate more neutral experiences of differences or variations in experiences across social locations that are not inherently oppressive. The plight of poor homosexuals may indeed reflect a multiple jeopardy that accrues at the intersection of the oppressive forces of heterosexism and capitalism. However, the interaction between gender and race reported here suggests that certain characteristics of South Asian communities are detrimental for the health of women and beneficial for the health of men. If patriarchal gender relations within South Asian families are culpable [41] then inequality by gender is clearly a factor here but race relations perhaps are not. The interaction between gender and class in turn points to the particularly heavy penalty paid by lower class men; here class inequality among men [24] may be more pertinent than gender relations between men and women. These provocative findings point to the importance of applying to health disparities in Canada a version or understanding of intersectionality theory that can accommodate intersections of different kinds and qualities.

The theory of "invisible intersectionality" has this potential. Valerie Purdie-Vaughns and Richard Eibach [42] argue that people with multiple subordinate-group identities who do not fit the prototypes of their constituent groups are "marginal members of marginal groups" who are relegated to positions of "acute social invisibility." While there are certainly disadvantages to holding multiple subordinate-group identities, they argue that there can be *advantages *to social invisibility in that marginal members of marginal groups may be able to elude the more active forms of oppression which are directed at "prototypical" members of marginal groups. The multiplicity of multiplicative possibilities described in my analyses begs for further investigation from an intersectional invisibility perspective. For example, characteristics of workplaces and occupations, health behaviors, residential segregation, experiences with systemic, institutional, and interpersonal discrimination, adherence to different norms of masculinity and femininity, and encounters with the health care system may identify advantages and disadvantages adhering to various complex social locations and explicate varying risks of poor health in Canada by intersecting axes of inequality. However, acknowledging with Weber and Parra-Medina [43] that intersectionality theory should focus on the social construction of complex identities in specific times and places and that survey data cannot explicate the ways in which relations of power operate in individual lives, some of these explanations may be amenable to investigation by way of survey research but others undoubtedly require other modes of investigation. Ethnographic investigation spanning interpersonal relations and institutional/structural arrangements may also be needed to substantiate and explicate the results described here.

## Conclusions

From an additive, non-intersectional perspective, poor self-rated health outcomes were reported by respondents claiming Aboriginal, Asian, or South Asian affiliations, lower class respondents, and bisexual respondents. However, from an intersectional perspective, each axis of inequality interacted significantly with at least one other: multiple jeopardy pertained to poor homosexuals and (possibly) South Asian women who were at an unexpectedly high risk of fair/poor self-rated health and mitigating effects were experienced by poor women and by poor Asians who were less likely than expected to report fair/poor health. I conclude from these varied results that the intersectionality theory best suited for explicating health inequalities in Canada should be theoretically capable of accommodating axis intersections of multiple kinds and qualities.

## Competing interests

The author declares that he has no competing interests.
